# Deciphering global gene expression and regulation strategy in *Escherichia coli* during carbon limitation

**DOI:** 10.1111/1751-7915.13343

**Published:** 2018-12-11

**Authors:** Zongjin Li, Qing Pan, Yunzhu Xiao, Xingxing Fang, Ruoping Shi, Chunxiang Fu, Antoine Danchin, Conghui You

**Affiliations:** ^1^ Shenzhen Key Laboratory of Microbial Genetic Engineering College of Life Sciences and Oceanology Shenzhen University 1066 Xueyuan Rd Shenzhen 518055 Guangdong P. R. China; ^2^ Shandong Provincial Key Laboratory of Energy Genetics Key Laboratory of Biofuels Qingdao Engineering Research Center of Biomass Resources and Environment Qingdao Institute of Bioenergy and Bioprocess Technology Chinese Academy of Sciences 189 Songling Rd Qingdao 266101 Shandong P. R.China; ^3^ Integromics Institute of Cardiometabolism and Nutrition Hôpital de la Pitié‐Salpêtrière 47 Boulevard de l'Hôpital 75013 Paris France; ^4^ School of Biomedical Sciences Li KaShing Faculty of Medicine Hong Kong University 21 Sassoon Road Pokfulam Hong Kong

## Abstract

Despite decades of studies meant to analyse the bacterial response to carbon limitation, we still miss a high‐resolution overview of the situation. All gene expression changes observed in such conditions cannot solely be accounted for by the global regulator Crp either free or bound to its effector, cyclic AMP. Here, for the first time, we evaluated the response of both CDS (protein‐coding sequence) and ncRNA (non‐coding RNA) genes to carbon limitation, revealed cellular functions of differentially expressed genes systematically, quantified the contribution of Crp‐cAMP and other factors to regulation and deciphered regulation strategies at a genomewide scale. Approximately one‐third of the differentially expressed genes we identified responded to Crp‐cAMP via its direct or indirect control, while the remaining genes were subject to growth rate‐dependent control or were controlled by other regulators, especially RpoS. Importantly, gene regulation mechanisms can be established by expression pattern studies. Here, we propose a comprehensive picture of how cells respond to carbon scarcity. The global regulation strategies thus exposed illustrate that the response of cell to carbon scarcity is not limited to maintaining sufficient carbon metabolism via cAMP signalling while the main response is to adjust metabolism to cope with a slow growth rate.

## Introduction

The enterobacterium *Escherichia coli* is used as a *chassis* in industry for the production of a wide variety of proteins or metabolites, but it is subject to frequent nutrient depletion during fermentation. A comprehensive exploration of the mechanisms which allow it to adapt to nutrient depletion has been undertaken in a considerable number of studies, generally focusing on carbon limitation (Oh *et al*., 2002; Gosset *et al*., 2004; Hua *et al*., 2004; Liu *et al*., 2005). By and large, an increased expression of genes involved in the catabolism of compounds made exclusively of carbon or of mixed atomic composition has been observed during carbon limitation in *E. coli*, with relatively few genes downregulated. This was paralleled with an activation of the cAMP signalling pathway (Makman and Sutherland, 1965; Perlman *et al*., 1969; Saier *et al*., 1976; Deutscher *et al*., 2006). cAMP binds to its receptor Crp, and the Crp‐cAMP complex activates the expression of many carbon catabolic genes (Kolb *et al*., 1993; Lawson *et al*., 2004). Besides Crp‐cAMP, several global regulators are also differentially expressed during carbon limitation (Oh *et al*., 2002; Liu *et al*., 2005). However, we miss a straightforward way to resolve their relative contribution to regulation. This is rendered particularly difficult because the cell growth rate slows down when the carbon supply becomes limiting, while this must have important consequences on gene expression. In particular, ribosome‐dependent protein synthesis is well known to be highly sensitive to growth rate‐dependent control (GRDC), using ppGpp signalling in particular (Hernandez and Bremer, 1990; Scott *et al*., 2010; Lemke *et al*., 2011), and this will obscure the contribution of other important regulatory features. As cases in point, several genes belonging to a variety of pathways were reported to respond also to GRDC (Pedersen *et al*., 1978; Jones *et al*., 1990; Pease *et al*., 2002; Rand *et al*., 2002). Here, we attempted to disentangle this conundrum by exploring the contribution of the Crp‐cAMP regulation to well‐defined patterns of carbon limitation.

By applying strand‐specific RNA‐Seq, we first measured the expression level of both CDS and ncRNA genes during carbon limitation, detected differentially expressed genes (DEGs), observed how their expression varied in families of consistent patterns, identified unannotated promoters accordingly and organized them in various functional categories. Next, by comparing the transcriptomes obtained during carbon limitation with the transcriptome of a cAMP‐signal deletion strain, we characterized genes that responded to Crp‐cAMP, were subject to GRDC or were controlled by other regulators. This allowed us to uncover gene regulation strategy at a genomewide scale. Finally, we showed that novel gene regulation mechanisms can be deciphered using our knowledge of their expression patterns.

## Results

### Quantitation and comparison of gene expression during carbon limitation

**Figure 1:**
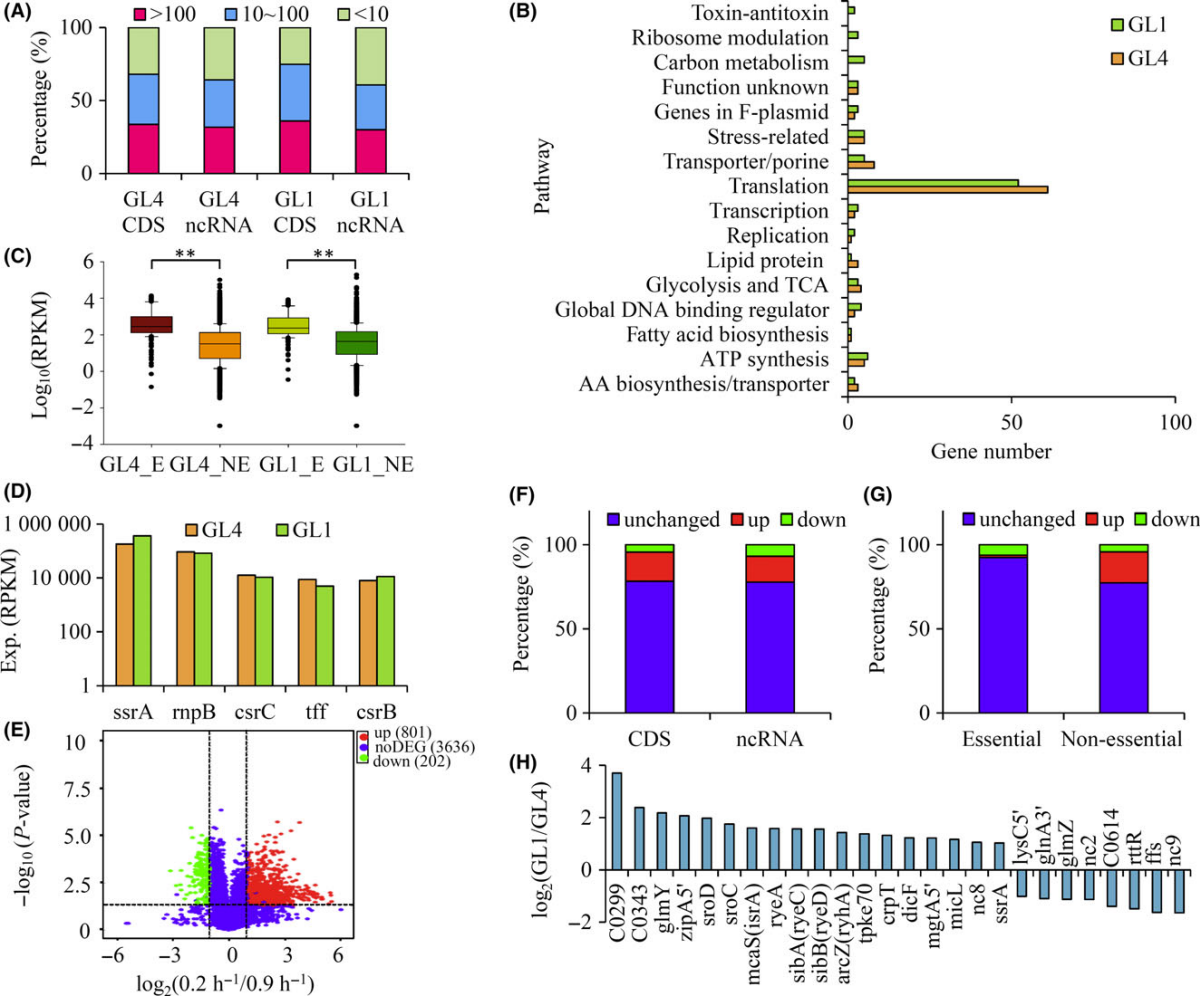
Gene expression levels and DEGs identified during carbon limitation. A. Percentage of genes with different expression levels. B. Function of top 100 highly expressed CDS genes. C. Comparing expression levels of essential and non‐essential genes. D. Expression levels of top five highly expressed ncRNA genes. E. Volcano map comparing transcriptomes of cells grown in carbon‐limited chemostat between growth rate of 0.2 and 0.9 h^−1^. Horizontal line points to *P*‐value = 0.05 on *y*‐axis, and vertical lines point to twofold cut‐off of the expression on *x*‐axis. Red dots: upregulated genes; green dots: downregulated genes; and blue dots: genes showed no changes (noDEG). F–G. Percentage of CDS or ncRNA genes (F) and essential or non‐essential genes (G) identified to be upregulated, downregulated or unchanged during carbon limitation. H. Fold changes of ncRNA during carbon limitation between growth rate of 0.2 and 0.9 h^−1^. GL4: carbon‐limited chemostat with a growth rate of 0.9 h^−1^; GL1: carbon‐limited chemostat with a growth rate of 0.2 h^−1^. GL4‐E, GL4‐NE, GL1‐E and GL1‐NE: essential (E) and non‐essential (NE) genes in the condition of GL4 or GL1. **P < 0.01 by Student *t*‐test.

To get a high‐resolution global response to carbon limitation, we analysed by RNA‐Seq the transcriptomes of a wild‐type *E. coli* K‐12 strain grown at defined growth rates in a carbon‐limited chemostat. We first monitored two transcriptomes, in a carbon‐rich condition (growth rate of 0.9 h^−1^) and in a severe carbon‐limited condition (growth rate of 0.2 h^−1^). In both transcriptomes, the expression level of both CDS and ncRNA genes was evaluated: one‐third was highly expressed (> 100 RPKM); one‐third was moderately expressed (10–100 RPKM); and one‐third was expressed at a low level (< 10 RPKM; Fig. 1A).

We focused on the top 100 highly expressed CDS genes in both transcriptomes (Fig. 1B). Not unexpectedly, more than half of those were ribosomal genes. The other top 100 genes were involved in various key metabolic pathways. In contrast to the picture displayed by the 0.9 h^−1^ transcriptome, several carbon metabolism genes, toxin and antitoxin genes and ribosome modulation genes belonged to the top 100 genes in the 0.2 h^−1^ transcriptome, revealing their importance during carbon limitation.

Interestingly, among the top 100 genes, 49 and 43 genes (respectively in the 0.9 h^−1^ and the 0.2 h^−1^ transcriptome) were also genes deemed essential (Baba *et al*., 2006), substantiating previous work that showed that essential genes, which make the major component of the core proteome (Yang *et al*., 2015), tend to be highly expressed (Acevedo‐Rocha *et al*., 2013). Indeed, we found that the expression level of all genes recognized as essential was significantly higher than that of the non‐essential genes (Fig. 1C).

The top five highly expressed ncRNA genes were *ssrA*,* rnpB*,* csrC*,* tff* and *csrB* in both transcriptomes (Fig. 1D). Similar to the highly expressed CDS genes, most of these ncRNA are known to be involved in important biological processes. SsrA, also called tmRNA, rescues unproductively stalled ribosomes (Withey and Friedman, 2003). It may be worth noticing that *ssrA* showed the highest expression level among all the CDS and ncRNA genes during all the conditions studied. The RnpB RNA is the ribonuclease P ribozyme that works in association with the RnpA protein (Kole and Altman, 1979), processing tRNA precursor molecules (Chang and Carbon, 1975). CsrC and CsrB modulate the activity of carbon storage regulator CsrA (Weilbacher *et al*., 2003; Perez‐Morales and Bustamante, 2016), which is a widely conserved protein that regulates carbohydrate metabolism. The only unexpected ncRNA gene was *tff* (Rivas *et al*., 2001), a gene located upstream of ribosome protein gene *rpsB* (Aseev *et al*., 2008). This RNA binds to ribosomal protein S2 and regulates its synthesis as well as that of elongation factors EF‐TuA and EF‐TuB (Fu *et al*., 2013).

We next characterized the expressed genes (DEGs) that differentiated the 0.2 h^−1^ transcriptome from the 0.9 h^−1^ transcriptome (Fig. 1E). 1003 DEGs, including 977 CDS genes and 26 ncRNA genes, were retained as significant with a twofold cut‐off (with a *P*‐value smaller than 0.05; Data S1).

Most of these DEGs (70–80%), whether ncRNAs or CDSs, were upregulated during carbon limitation (Fig. 1F). Interestingly, essential genes were distinct from non‐essential genes in two aspects (Fig. 1G): less essential genes were differentially expressed (~7% versus ~20%), while most differentially expressed essential genes (~80%) were downregulated instead of upregulated during carbon limitation. The changes in ncRNA differential expression are shown in Fig. 1H. C0299 (now CsrE) and nc9 (now HurI) were the two that showed the biggest changes among the up‐ and downregulated ncRNA respectively.

### Gene expression patterns parallel growth rates during carbon limitation

**Figure 2:**
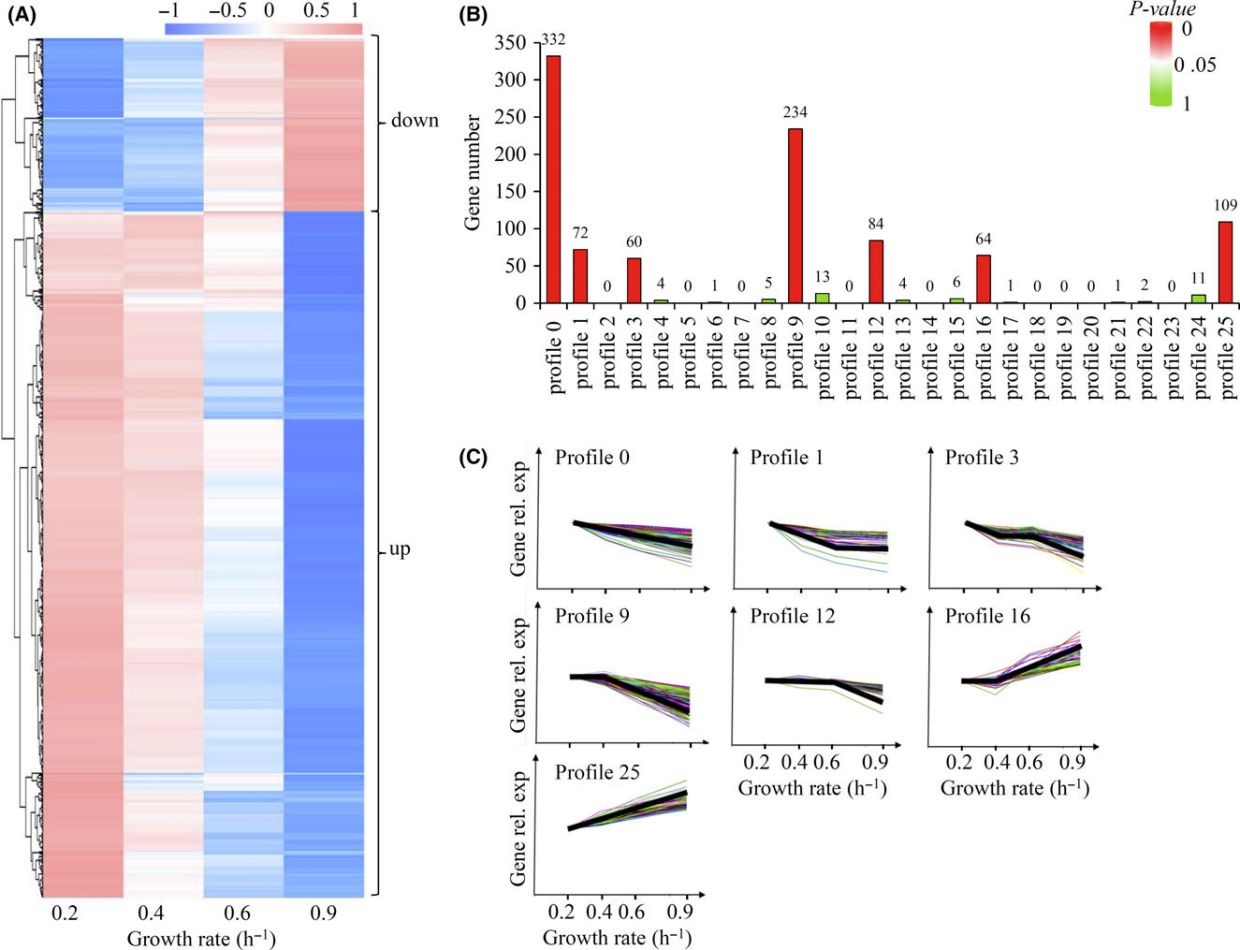
Gene expression patterns following growth rates during carbon limitation. A. Heat map of expression changes of up‐ and downregulated DEGs following growth rates. B. Classification of gene expression pattern changes following growth rates. Profiles with *P*‐value < 0.05 and *P*‐value > 0.05 are shown in red and green respectively. C. Expression changes of genes in significantly enriched profiles following growth rates. Coloured lines exhibit genes expression changes, and thick black lines indicate the trend of the whole profile to guide the eye.

In order to get an overview of how these DEGs changed during carbon limitation, we monitored additional transcriptomes in moderate carbon‐limited conditions (growth rate of 0.4 and 0.6 h^−1^) and gene expression at the four growth rates was plotted in a heat map (Fig. 2A). To further classify gene expression patterns following growth rates, we carried out cluster analysis (Ernst and Bar‐Joseph, 2006). Following this breakdown, the DEGs were clustered into 17 expression patterns that we called “profiles” (Fig. 2B, Data S2). Seven profiles with *P*‐value < 0.05 were significantly enriched profiles (Fig. 2C). Profile 0 and profile 25 respectively carried the largest number of upregulated and downregulated genes. The DEGs in both profiles changed continuously with growth rates but in opposite directions, supporting previous findings on several selected genes (Scott *et al*., 2010; You *et al*., 2013). The DEGs in the remaining profiles all showed expression plateaus at various growth rates (Fig. 2C, Fig. S1).

### Identification of new promoters by comparison of gene expression levels and expression patterns

**Figure 3:**
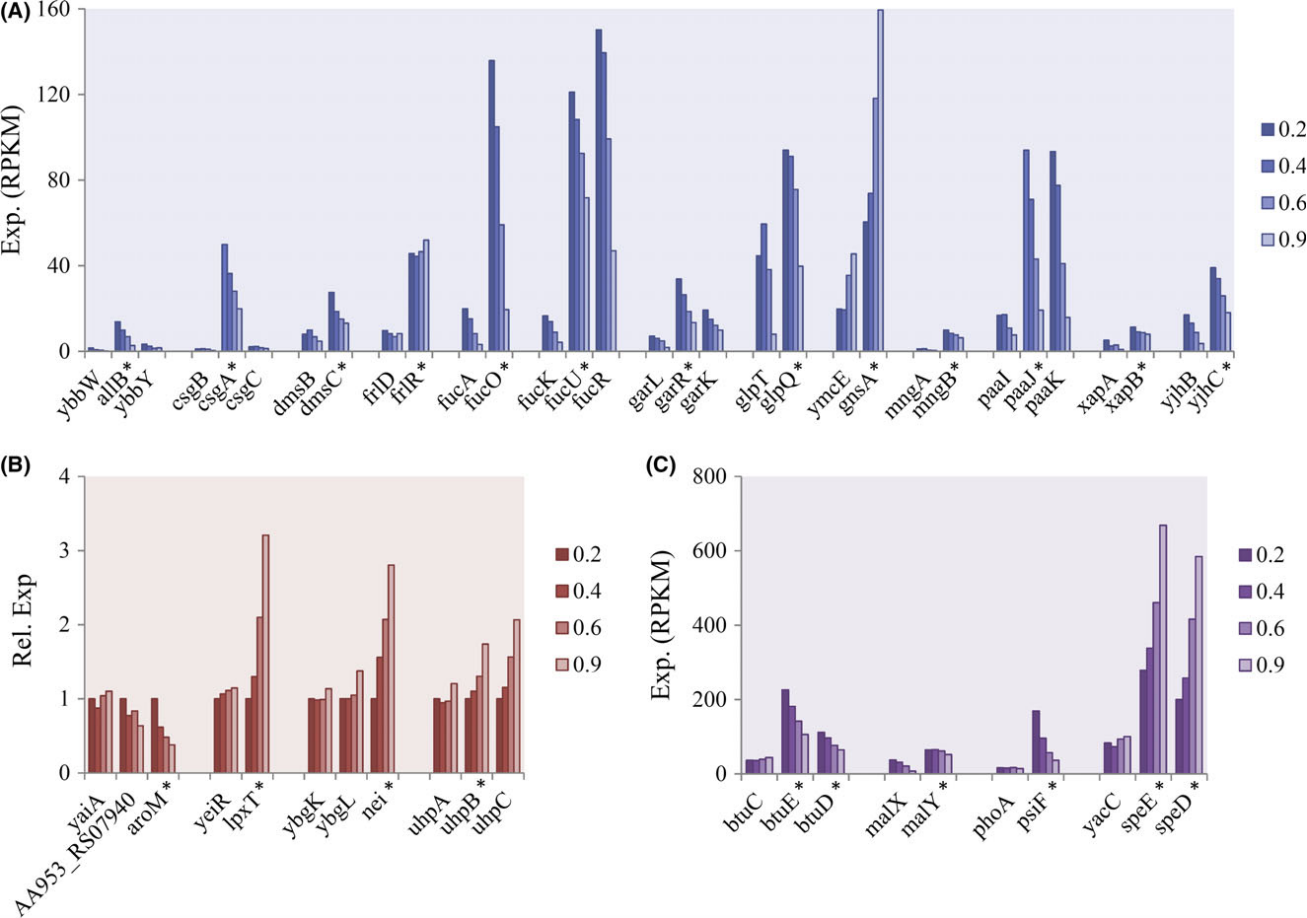
Expression of genes in operons with putative internal promoters. A–C. Expression (A, C) or relative expression (B) of genes carrying putative promoters (with star), their corresponding upstream gene or downstream gene within the same operon at the four growth rates (0.2, 0.4, 0.6 and 0.9 h^−1^). In (A), expression of the gene carrying putative promoter is much higher that its upstream gene. In (B), expression pattern of the gene carrying putative promoter following growth rate is different as compared to its upstream gene. Gene expression at 0.2 h^−1^ was normalized to 1, and its relative expression at the other growth conditions was determined relative to this value. In (C), expression of the gene carrying putative promoter is much higher and it also shows different expression patterns as compared to its upstream gene.

Genomewide knowledge of gene expression levels has been used to study operon architecture of *E. coli* grown in log and lag phase. Differential gene expression within operons suggested the existence of internal promoters (Conway *et al*., 2014; Gama‐Castro *et al*., 2016). Here by further integrating the knowledge of gene expression patterns following growth rate variation during carbon limitation, we identified 23 genes carrying new promoters that were still unannotated (Fig. 3). The selection criteria we used were the following: if the expression in an operon of a downstream gene B was higher than its upstream gene A (criterion 1), or if the expression patterns of downstream gene B and upstream gene A were different (criterion 2), gene B could be expressed by an internal promoter besides the operon promoter. Genes matching criterion 1 or criterion 2 are shown in Fig. 3A and B respectively. Genes matching both criterion 1 and criterion 2 were also identified (Fig. 3C). We next characterized the transcriptional start site of these putative new promoters using 5′ RACE assays, and the corresponding promoter regions were predicted (Table S1).

### Cellular functions of DEGs identified

**Figure 4:**
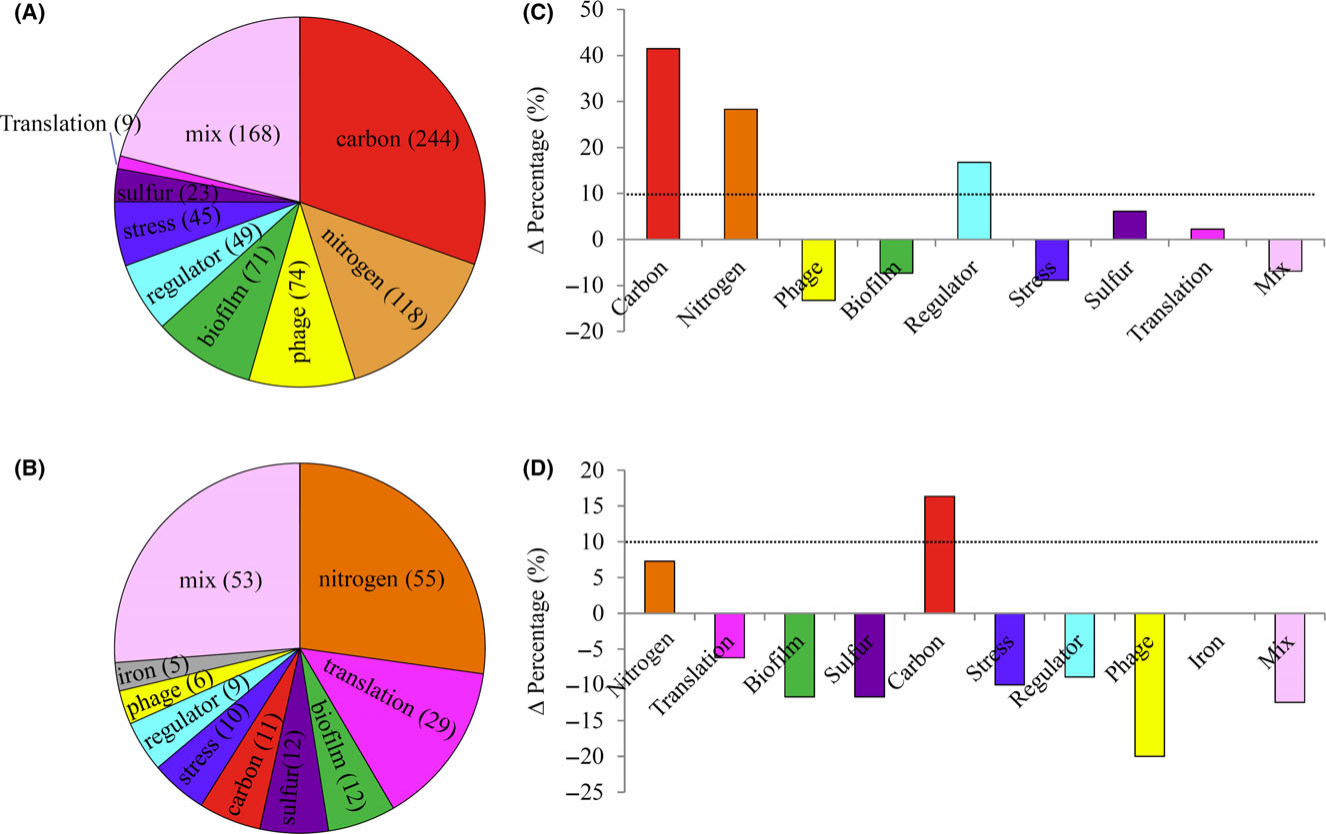
Functional categories of DEGs identified. A–B. Pie chart shows percentages of genes in each functional category. Gene number of each category was shown in the brackets. C–D. The Crp‐cAMP binding percentage in each category minus the Crp‐cAMP binding percentage of the whole genome (Δ percentage). The dash lines point to Δ percentage = 10%. A and (C):upregulated genes; (B) and (D):downregulated genes.

The pace of accurate annotation of the *E. coli* genome has slowly decreased and has not been improved for the past 5 years or so. In order to gain insight into the processes we were investigating, we updated the annotations of approximately 200 y‐genes (genes with no attributed function and EcoGene Y‐entries) in Data S1, based on published literature. The functions of nearly 90% of all the DEGs identified were discovered accordingly. Remarkably, both up‐ and downregulated genes could be organized in a consistent fashion of various categories according to their functions. Gene numbers in each category are shown in Fig. 4.

In consistent with the literature (Kolb *et al*., 1993; Zheng *et al*., 2004; Deutscher, 2008), nearly half of the upregulated genes appeared in ‘carbon’ and ‘nitrogen’ categories, which contained genes functioned in catabolism of compounds made exclusively of carbon ‘carbon’ and made of nitrogen atom composed carbon sources ‘nitrogen’, including amino acid, purine and pyrimidine, and amino sugar. (Fig. 4A). Genes related to biofilm formation (Jackson *et al*., 2002; Franchini *et al*., 2015; ‘biofilm’), various stress (Franchini *et al*., 2015; ‘stress’) and transcription factors (TFs; Liu *et al*., 2005; Oh *et al*., 2002; ‘regulator’) had been characterized accordingly. The category of ‘phage’, which included various prophage genes, the category of ‘sulfur’, which included genes related to sulfur metabolism essentially around glutathione repair system, and the category of ‘translation’, which included genes related to ribosome association, ribosome modulation factor and tmRNA, were identified for the first time. The remaining genes labelled ‘mix’ mainly included aerobic and anaerobic respiration genes, putative transporters and function unannotated genes.

Supporting our previous findings (You *et al*., 2013), nearly half of the downregulated genes appeared in ‘nitrogen’ and ‘translation’ categories, which functioned in biosynthesis of amino acid, purine and pyrimidine (‘nitrogen’), and protein translation, tRNA and rRNA modification (‘translation’) (Fig. 4B). Genes in ‘biofilm’ were related to pili formation and c‐di‐GMP metabolism, a signalling molecular controlling biofilm formation (Boyd and O'Toole, 2012; Opoku‐Temeng and Sintim, 2017). The category of ‘sulfur’, mainly contained sulfate transporter, and the category of ‘carbon’ included carbon source efflux genes and phosphate‐sugar metabolism genes. Several TFs genes ‘regulator’, prophage genes ‘phage’ and iron/haem metabolic genes ‘iron’ were also identified. There were still remaining genes labelled ‘mix’ and most of their function needed to be further discovered by novel annotation.

We then explored Crp‐cAMP regulation on the genes in each category *in silico*. Based on RegulonDB (Gama‐Castro *et al*., 2016), Ecocyc (Keseler *et al*., 2017) and recent studies of Crp‐cAMP regulon (Zheng *et al*., 2004; Shimada *et al*., 2011), the Crp‐cAMP binding percentage in the genome is 20% (928/4639). For the upregulated genes, we found genes with promoters binding to Crp‐cAMP enriched in ‘carbon’, ‘nitrogen’ and ‘regulator’ categories because Crp‐cAMP binding percentage in each of the three categories was > 10% higher as compared to 20% (Fig. 4C). Similarly, for the downregulated genes, genes with Crp‐cAMP binding sites in their promoter regions were enriched in the ‘carbon’ category (Fig. 4D). Given that most genes were under coordinated regulation by several TFs, the existence of Crp‐cAMP binding site on the promoter region of a gene does not necessarily prove that this gene will respond to Crp‐cAMP during carbon limitation. But this analysis indicated that Crp‐cAMP had more chances to take the priority role on the regulation of catabolism as compared to genes in other categories, which supported the well‐known function of Crp‐cAMP (Kolb *et al*., 1993; Zheng *et al*., 2004; Deutscher, 2008). Moreover, Crp‐cAMP and Crp‐cAMP‐regulated TFs may compose a regulatory hierarchy on the DEGs identified here.

### Uncovering gene regulation strategy at a genomewide scale

**Figure 5:**
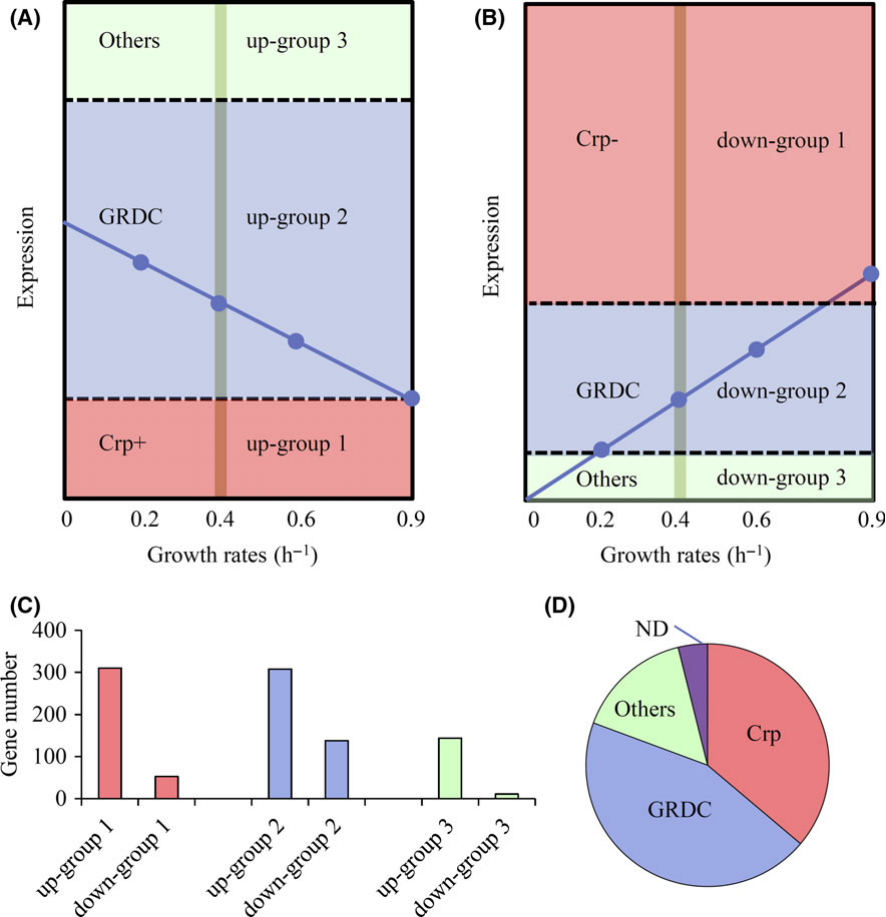
Identification of global regulation mechanisms. A–B. Cartoons of criteria used to identify genes responded to Crp‐cAMP (Crp−: repressed by Crp‐cAMP; Crp+: activated by Crp‐cAMP), subjected to GRDC or controlled by other regulators (others) for the upregulated (A) or downregulated (B) genes during carbon limitation. The blue dots and lines represent expression of example genes at the four growth rates. The shaded vertical lines show possible expression value of the genes in CY104. (C) Gene numbers in each regulatory group. D. Pie chart shows percentages of genes responded to Crp‐cAMP, subjected to GRDC or controlled by other regulators. ND: genes not determined.

We compared the transcriptomes of a *cyaA* and *crp* double deletion mutant, CY104, grown in glucose at a growth rate of 0.4 h^−1^, to transcriptome of the wild‐type strain grown in glucose‐limited chemostat at the same growth rate. Integrating the knowledge of gene expression change trends following growth rate, we exposed gene regulation strategies at a genomewide scale. We identified six groups of DEGs: those which responded to Crp‐cAMP (up‐group 1 and down‐group 1); those subject to GRDC (up‐group 2 and down‐group 2); and those controlled by other regulators (up‐group 3 and down‐group 3; Data S3). The grouping procedure is illustrated in Fig. 5A–B, and the number of genes belonging to each group is shown in Fig. 5C.

The expression in strain CY104 (Exp104) of the upregulated genes activated by Crp‐cAMP belonging to up‐group 1 was significantly lower [Exp104/Exp4 < 0.5, using a twofold cut‐off (with a *P*‐value smaller than 0.05)] than their expression in the wild‐type strain (Exp4). In contrast, for the downregulated genes repressed by Crp‐cAMP belonging to down‐group 1, Exp104/Exp4 is > 2, showing that their expression in the double deletion mutant was significantly higher than in the wild‐type strain. The values of Exp104/Exp4 in these two groups would locate them in the region shaded in red in Fig. 5A–B. Taken together, the genes in these two groups made 36% of all DEGs (Fig. 5D).

**Figure 6:**
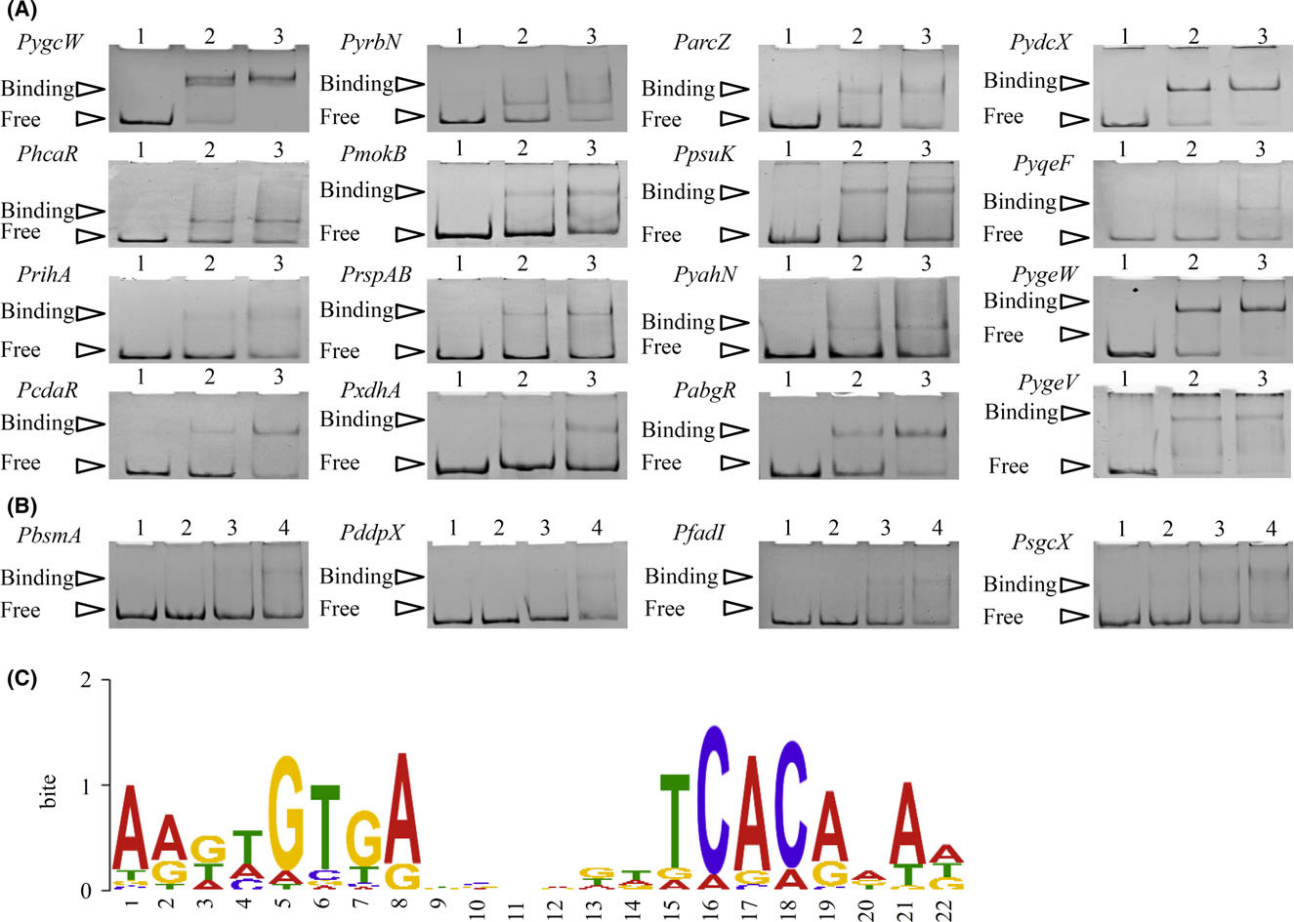
New promoters identified binding to Crp‐cAMP. A–B. EMSA of Crp‐cAMP binding to promoters. Fifty nano molar of each promoter fragment was incubated as described in Materials and Methods, alone (lane 1 in A–B), with 300 nM CRP (lane 2 in A–B), with 600 nM CRP (lane 3 in A–B) and with 1200 nM CRP (lane 4 in B). Two to six independent repeats were performed for each assay. (C) The logo is the consensus sequence analysis of predicted Crp‐cAMP binding sites of the genes identified (Table S2) by the MEME Suite (http://meme-suite.org/).

As expected, most CDS genes responding primarily to Crp‐cAMP code for catabolic processes or for transporters of various carbon sources (Kolb *et al*., 1993; Zheng *et al*., 2004; Deutscher, 2008). Six ncRNA genes showed up in up‐group 1. 54% of the genes belonging to up‐group 1% and 25% of the genes belonging to down‐group 1 have been previously reported to be regulated by Crp‐cAMP directly. For the remaining genes, not previously known to be regulated by Crp, we performed electrophoretic mobility shift assays (EMSA) on a random selection of 63 genes. We found that the promoters of 34 genes, including one ncRNA gene (*arcZ*), belonging to these two groups bound Crp‐cAMP (Fig. 6). This makes ~50% of the genes we assayed. The putative Crp‐cAMP binding site of each promoter was predicted *in silico* (Table S2). It matched the consensus recognition sequences of Crp‐cAMP (TGTGAN_6_TCACA; Fig. 6C; Busby and Ebright, 1999; Ishihama, 2010).

Besides direct regulation by Crp‐cAMP, the genes could be regulated by Crp‐cAMP indirectly. As shown in Table 1, 21 DEGs coding TFs appeared in up‐group 1. Within the 21 genes, the promoters of 17 genes showed direct binding to Crp‐cAMP. Therefore, genes regulated primarily by these regulators may respond to Crp‐cAMP through an indirect effect. For example, the promoter of *cdaR* (coding for a transcriptional regulator) bound Crp‐cAMP (Fig. 6A), and one of the CdaR‐regulated genes, *garD*, also appeared in up‐group 1. That *garD* showed up in this group could be an indirect effect of Crp‐cAMP. The same could account for the fact that MalT‐regulated *malPQ* or GlcC‐regulated *glcAB* belonged to this group. Interestingly, we found that many of these regulators need to bind to a metabolite ligand to be activated (Table 1). Here comes a question: Why these regulators need to be activated by Crp‐cAMP? The possible mechanism we proposed is that when it is difficult to have enough metabolite ligand during carbon limitation, Crp‐cAMP increase the expression of regulator protein directly to form more active regulator–ligand complex in order to induce the expression of catabolic genes.

**Table 1:** DEGs coding TFs

Gene	Ligand or cofactor^a^	Classification^b^	Crp‐cAMP^c^	Reference
*malT*	Maltotriose	Up‐group 1	Yes	RegulonDB
*rhaS*	L‐Rhamnose	Up‐group 1	Yes	RegulonDB
*rhaR*	L‐Rhamnose	Up‐group 1	Yes	RegulonDB
*melR*	Melibiose	Up‐group 1	Yes	RegulonDB
*dcuR*	DcuS‐fumarate	Up‐group 1	Yes	RegulonDB
*xlnR*(*yjhI*)	Xylonate	Up‐group 1	Yes	RegulonDB
*lgoR*	L‐Galactonate	Up‐group 1	Yes	Ecocyc
*araC*	D‐Arabinose	Up‐group 1	Yes	RegulonDB
*mhpR*	3‐Hydroxyphenylpropionate + phenylpropionate	Up‐group 1	Yes	RegulonDB
*feaR*	Aromatic aldehyde (after deamination)	Up‐group 1	Yes	RegulonDB
*lsrR*	AI‐2	Up‐group 1	Yes	RegulonDB
*glcC*	Glycolate	Up‐group 1	Yes	RegulonDB
*tdcA*	Threonine/serine	Up‐group 1	Yes	RegulonDB
*ybaE*	\	Up‐group 1	No	This work
*mtfA*	Mlc	Up‐group 1	No	This work
*atoS*	Acetoacetate	Up‐group 1	No	This work
*atoC*	AtoS+acetoacetate	Up‐group 1	No	This work
*cdaR*	D‐Glycerate, glucarate or galactarate	Up‐group 1	Yes	This work
*abgR*	p‐Aminobenzoyl‐glutamate	Up‐group 1	Yes	This work
*hcaR*	3‐Phenylpropionate	Up‐group 1	Yes	This work
*ygeV*	Sigma 54 factor	Up‐group 1	Yes	This work
*bolA*	Glutaredoxin	Up‐group 2	No	This work
*rssB*	RpoS	Up‐group 2	No	This work
*pspC*	\	Up‐group 2	No	This work
*paaX*	Phenylacetyl‐CoA	Up‐group 2	No	This work
*ydcI*	\	Up‐group 2	No	This work
*ada*	\	Up‐group 2	No	This work
*eutR*	B12 + ethanolamine	Up‐group 2	No	This work
*fhlA*	\	Up‐group 2	No	This work
*lldR*	L‐Lactate	Up‐group 2	No	This work
*dmlR*	D/L‐Malate	Up‐group 2	No	This work
*csiR*	\	Up‐group 2	No	This work
*ompR*	EnvZ	Up‐group 2	Yes	RegulonDB
*sgcR*	\	Up‐group 2	Yes	This work
*arcA*	\	Up‐group 2	Yes	Zheng *et al*. (2004)^d^
*allS*	Allantoin	Up‐group 2	Yes	This work
*ybhD*	\	Up‐group 2	Yes	Zheng *et al*. (2004)^d^
*mlrA*	YegE/YhjH and YdaM/YciR	Up‐group 2	Yes	This work
*rseA*	Anti‐sigma E	Up‐group 2	Yes	RegulonDB
*rpoS_C*	\	Up‐group 2	Yes	RegulonDB
*yiaG*	\	Up‐group 3	No	This work
*yjbR*	\	Up‐group 3	No	This work
*adiY*	\	Up‐group 3	No	This work
*yagP*	\	Up‐group 3	No	This work
*bssR*	\	Up‐group 3	No	This work
*ariR*	RcsB	Up‐group 3	No	This work
*yeeY*	\	Up‐group 3	No	This work
*rcsA*	RcsB	Up‐group 3	No	This work
*gadE*	alone or RcsB	Up‐group 3	Yes	RegulonDB
*matA*(*ecpR*)	\	Down‐group 1	No	This work
*iraM*	RpoS	Down‐group 1	No	This work
*cnu*	StpA	Down‐group 1	No	This work
*mgrB*	PhoQ	Down‐group 1	No	This work
*uhpC*	UhpB‐glucose‐6‐P	Down‐group 2	No	This work
*crl*	RpoS	Down‐group 2	No	This work
*stpA*	\	Down‐group 2	No	This work
*mprA*(*emrR*)	Uncouplers such as dinitrophenol	Down‐group 2	No	This work
*fis*	\	Down‐group 2	Yes	RegulonDB

**a**. Ligand or cofactor that this regulator binds to.

**b**. Classification of genes basing on their regulatory strategies in Data S4.

**c**.’Yes’ means the promoter of the gene binds to Crp‐cAMP directly. ‘No’ means the promoter of the gene does not bind to Crp‐cAMP basing on EMSA assay of this work.

**d**. Zheng *et al*. (2004).

**Figure 7:**
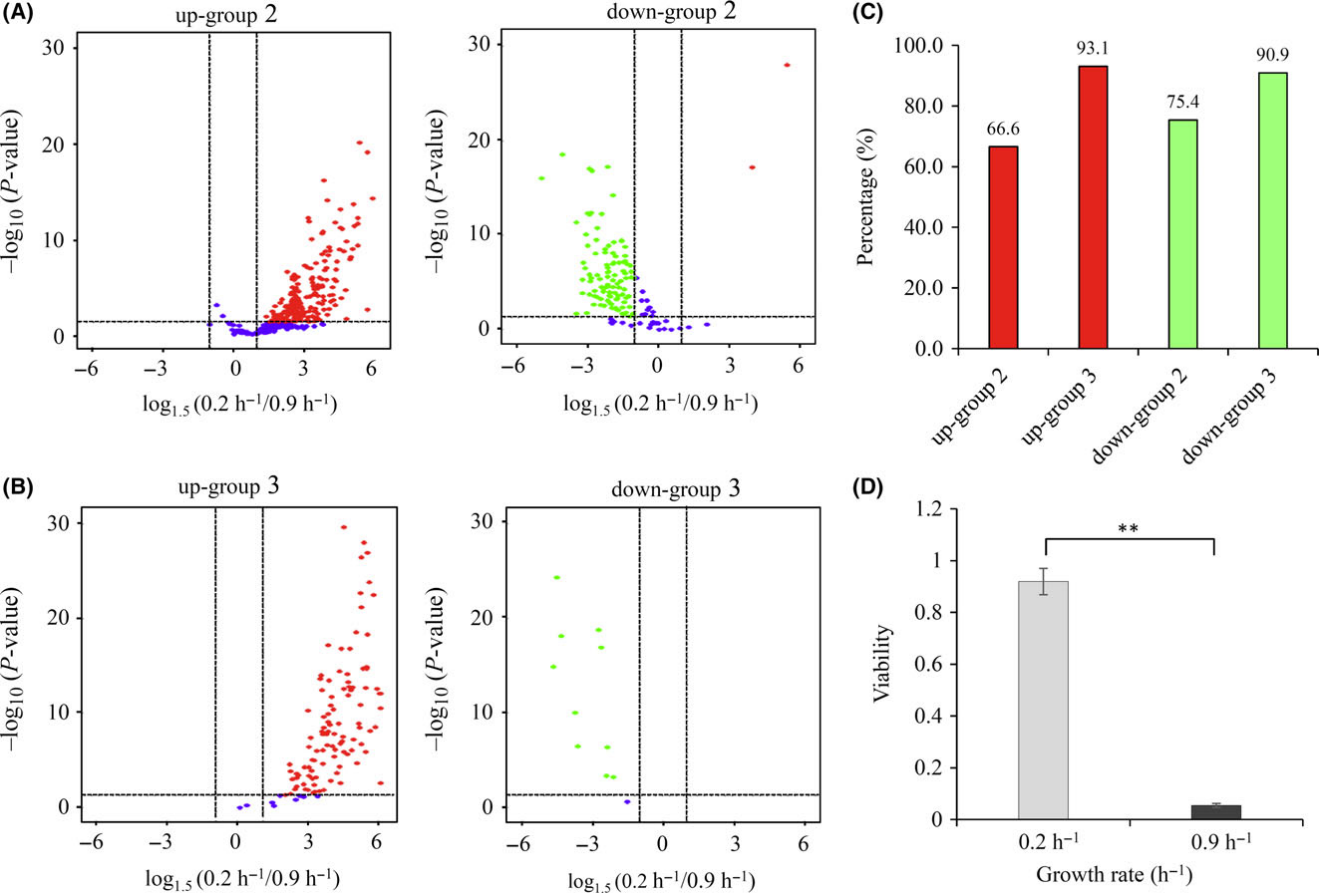
Validation of genes subjected to GRDC or controlled by other regulators. A–B. Volcano map comparing the expression of genes in down‐group 2, up‐group 2 down‐group 3 and up‐group 3 during nitrogen limitation between growth rate of 0.2 and 0.9 h^−1^. Horizontal line points to *P*‐value = 0.05 on *y*‐axis, and vertical lines point to 1.5‐fold cut‐off of the expression on *x*‐axis. Red dots indicate upregulated genes, green dots indicate downregulated genes, and blue dots indicate genes showed no changes. C. Percentage of genes in these four groups that similarly down‐ or upregulated during nitrogen limitation. D. Viability comparison of cells during carbon limitation at a growth rate of 0.2 or 0.9 h^−1^ challenged with oxidative stress. Viability was calculated as percentage of CFUs of cells treated with stress versus untreated cells. The data are presented as the mean ± SD of two independent experiments, and **P < 0.01 by Student's *t*‐test.

The expression of genes subject to GRDC that belong to up‐group 2 and down‐group 2 did not display differences between CY104 and the wild type [0.5 < Exp104/Exp4 < 2, using a twofold cut‐off (shaded in blue in Fig. 5A–B)] when grown at the same growth rate (Exp4). Taken together, the genes belonging to both groups occupied 44% of the DEGs (Fig. 5D). Ten and eight ncRNA genes showed up in up‐group 2 and down‐group 2 respectively. To make out the way they were co‐regulated, we studied the expression of these genes in a nitrogen‐limited chemostat at growth rates of 0.2 and 0.9 h^−1^. If the growth rate took the major role, they would be expected to respond to nitrogen limitation similarly. To be sure, most of these genes (75% of the genes belonging to down‐group 2 and 67% of the genes belonging to up‐group 2) were differentially expressed, with a > 1.5‐fold threshold and *P*‐value < 0.05 during nitrogen limitation (Fig. 7A and C). The list of the genes responding to nitrogen limitation is reported in Data S4.

The downregulated genes subjected to GRDC included genes encoding ribosomal proteins (*rplA*,* rplI*,* rplK*,* rplL*,* rplM*,* rplU*,* rpmA*,* rpmB*,* rpmG*,* rpmH*,* rpsI*,* rpsT*,* rpsU*), in line with the linear correlation known to exist between growth rate and cellular ribosome content (Scott *et al*., 2010). Consistent with its growth rate‐dependent expression, *fis*, encoding the Fis transcriptional regulator, also came out in this group (Mallik *et al*., 2006; Table 1). A variety of other genes related to translation was also found, including tRNA modification genes (*dusC*,* queA*,* thiI*), a rRNA modification gene (*rlmG*) and a ribosomal protein modification gene (*ycaO*) required for the efficient *in vivo* RimO‐dependent beta‐methylthiolation of ribosomal protein S12. Many other genes related to general biosynthesis processes, including amino acid biosynthetic genes (*argA*,* argCBH*,* argD*,* argG*,* cysM*,* dapB*,* dapD*,* gapA*,* ilvC*,* metE*,* serB*), amino acid transporter genes (*artJ*,* lysP*,* pheP*,* proVW*), and purine and pyrimidine biosynthetic or salvage genes (*apt*,* gsk*,* pyrE*,* uraA*). Consistent with our findings, a recent report showed a decreased expression of amino acid and nucleotide synthesis enzymes during a nutrient downshift caused by growth rate slowing down (Erickson *et al*., 2017). Interestingly, amino acid catabolic genes (*astCADBE*) and genes functioning in purine and pyrimidine catabolism (*cdd*,* deoA*,* deoB*,* xapA*) were subjected to GRDC but upregulated. Taken together, these results suggest a decreased amino acid and nucleotide synthesis rate at low growth rate, providing a molecular basis for the growth rate‐dependent changes of the cellular protein, RNA and DNA content (Bremer and Dennis, 1996). The remaining upregulated genes subject to GRDC operated in diverse pathways. For example, there were genes involved in pyruvate metabolism (*hchA*,* pflD*,* poxB*,* ppsA*,* ybiW*), glyoxylate and dicarboxylate metabolism (*acnA*,* gcl*,* glcF* and *hyi*) and oxidative phosphorylation (*cydABX*). Consistent with our finding, several non‐ribosomal genes have been reported to be subject to GRDC (Pedersen *et al*., 1978; Jones *et al*., 1990; Pease *et al*., 2002; Rand *et al*., 2002). We extended here considerably this observation at a high‐resolution genomewide scale.

The prevailing view assumed that biosynthetic pathways were not part of the Crp‐cAMP regulon (Franchini *et al*., 2015). Yet, except for ppGpp‐controlled ribosome genes, the signals connecting the other genes subject to GRDC remain elusive. The regulatory roles of the 24 TFs genes shown in these two groups (Table 1) would be the first candidates to explore using a systematic method reported recently (Gao *et al*., 2018).

Expression of the upregulated genes belonging to up‐group 3 in a cAMP signalling deletion mutant was significantly higher [Exp104/Exp4>2 (with a *P*‐value smaller than 0.05, shaded in green at Fig. 5A)] than in a wild‐type strain. These genes could be repressed by Crp‐cAMP. However, their expression increased during carbon limitation when their growth rate was reduced showing that they could be primarily activated by other regulators. The same line of reasoning, acting in reverse, could be applied to expression of the downregulated genes controlled by other regulators belonging to down‐group 3 [Exp104/Exp4 < 0.5 (with a *P*‐value smaller than 0.05, shaded in green at Fig. 5B)]. The genes in both groups occupied 15% of all DEGs (Fig. 5D). Interestingly, we found that 93% of the genes belonging to up‐group 3 were upregulated whereas 91% of the genes belonging down‐group 3 were downregulated during nitrogen limitation (Fig. 7B and C). This is consistent with the idea that signal(s) primarily controlling the expression of genes in these two groups during carbon limitation might not be derived from the carbon source per se but could originate from the low growth rate. The genes of both nitrogen limitation responding groups are listed in Data S4.

Owing to their increased expression of oxidative stress‐induced genes (*katE*,* sodC*,* osmC*), cells grown in carbon‐limited conditions were more resistant to oxidative stress than those grown in carbon‐rich conditions (Fig. 7D). We noticed that 48 genes present in up‐group 3, including these three genes, reported to be regulated by RpoS, suggesting that it may be involved as a regulator. Consistent with a previous study (Notley and Ferenci, 1996), the increased expression of *rpoS* during carbon limitation was also identified in our RNA‐Seq data. Given that *rpoS* appeared in up‐group 2, these 48 genes could be controlled coordinately by *rpoS* and other factors. As discussed with Crp‐cAMP, some genes in this group could be regulated by RpoS indirectly. RcsA is a case in point (Table 1). Besides autoactivation (Ebel and Trempy, 1999), *rcsA* was activated by GadE (Hommais *et al*., 2004) and *gadE* was one of the 48 genes controlled by RpoS (Patten *et al*., 2004; Table 1). The crucial DNA‐binding regulator for Rcs regulon is RcsA together with RcsB (Ebel and Trempy, 1999). Although *rcsB* did not change, the expression of *rcsA* showed a 3.5‐fold increase during carbon limitation. Rcs regulon members (Hagiwara *et al*., 2003; *wcaA*,* wcaD*,* wcaE*,* bdm‐rpsV*,* spy*,* yghA*,* glxO*,* yjbJ*) appeared in this group. The roles of other TFs genes that appeared in this group (Table 1) needed to be further explored. Additionally, Fur could be one of the primary regulators controlling some of the genes in down‐group 3 as four of the 11 genes (*fepE*,* cirA*,* fiu*,* ybiX*) in this group were reported to be repressed by Fur (Zhang *et al*., 2005; Seo *et al*., 2014).

### The knowledge of gene expression patterns reveals regulation strategies

**Figure 8:**
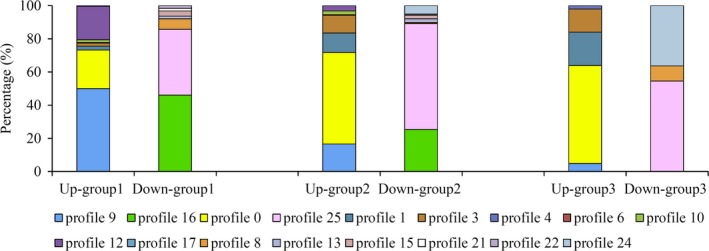
Expression profiles distribution in each regulatory group. In each regulatory group, percentage of genes in each expression profile was shown.

Will genes in each of these six regulatory groups show specific expression patterns? To answer this question, we studied the gene expression profile distribution of the genes in each one of the six groups previously described. We found that the genes regulated by Crp‐cAMP could be distinguished from genes regulated alternatively basing on their distinct expression patterns (Fig. 8). The dominant growth rate‐dependent expression patterns of the genes responding to Crp‐cAMP were profile 9 and profile 16. Both levelled off at 0.4 and 0.2 h^−1^. The major expression patterns of the genes subject to GRDC or controlled by other regulators were profile 0 and profile 25. They changed continuously when growth rates varied from 0.2 to 0.9 h^−1^. This provides us with a remarkable predictive power of the expression pattern of a gene in relation to its regulators. A gene exhibiting an expression plateau at 0.4 and 0.2 h^−1^ had more chances to be regulated by Crp‐cAMP, whereas a gene showing a continuous change had more chances to be subject to GRDC or to be controlled by other regulators.

## Discussion

With the help of strand‐specific RNA sequencing of bacteria grown in diverse conditions, we were able to get a high‐resolution picture of global genomic expression during carbon limitation. Strikingly, similar responses of CDS genes and ncRNA genes were noticed: in various carbon‐limited conditions, the expression of whole genome CDS and ncRNA genes varied with the same order of magnitude (Fig. 1A). Highly expressed CDS genes and ncRNA genes tend to dominate (Fig. 1B and D). By contrast, most DEGs of CDS and ncRNA genes showed an increased instead of a decreased expression during carbon limitation (Fig. 1F). In a further development, this study allowed us to quantify the dynamic changes of DEGs following growth rates at a much higher resolution than that of a recent proteomics study (Schmidt *et al*., 2016) and of a study reported 40 years ago (Pedersen *et al*., 1978; Fig. 2C, Fig. S1). This high resolution allowed us to explore in‐depth gene expression levels and expression patterns of the whole set of genes, while discovering previously unannotated promoters (Fig. 3, Table S1).

With our updated gene annotations, a comprehensive picture of how cells respond to carbon scarcity systematically is exposed (Fig. 4). In order to maintain sufficient carbon supply, cells induced the expression of catabolic enzymes of various compounds (Kolb *et al*., 1993; Zheng *et al*., 2004; Deutscher, 2008) and repress the expression of carbon source efflux enzymes simultaneously. However, the decreased growth rate during carbon limitation indicates that cells fail to maintain optimal carbon metabolism. Accordingly, cells decreased sulfur transporter genes to lower sulfur supply in order to match the decreased carbon influx (You *et al*., 2013; Hermsen *et al*., 2015). To further cope with the low growth rate, cells decreased the expression of biosynthesis genes such as ribosomal genes and amino acid biosynthesis genes (You *et al*., 2013; Erickson *et al*., 2017). Meanwhile, slow growth causes many stresses (Battesti *et al*., 2011) as compared to optimal growth with sufficient carbon supply which can be concluded from the DEGs known to be induced by various stress. And cells worked coordinately to survive through affecting biofilm formation genes (Nadell *et al*., 2009). Moreover, we found interesting genes related to age during slow growth, including the induced glutathione repair system (Danchin, 2018), suggesting a possible correlation of slow growth and cell ageing. One more interesting finding is the changed prophage genes during carbon limitation, and the physiological roles of these genes need to be further explored.

We quantified here the contribution of Crp‐cAMP, growth rate as well as other factors as RpoS to regulation during carbon limitation while uncovering regulation strategies at a genomewide scale (Fig. 5, Data S3). The genes responding to Crp‐cAMP make approximately one‐third of all the DEGs we identified, while nearly two‐thirds of all the DEGs is subject to GRDC or controlled by other regulators, such as RpoS (Fig. 5D). Most DEGs responding to Crp‐cAMP during carbon limitation are carbon catabolic genes and carbon source transporter genes (Kolb *et al*., 1993; Zheng *et al*., 2004; Deutscher, 2008). Crp‐cAMP together with TFs under direct regulation of Crp‐cAMP (Table 1) composed a regulatory hierarchy on these genes. Given that most genes subjected to GRDC or controlled by other regulators also respond to nitrogen limitation (Fig. 7A–C), the signal(s) controlling these genes are likely to monitor growth rates (in particular when growth is slow) or stress (Notley and Ferenci, 1996; Liu *et al*., 2005). This agrees with regulators such as ppGpp and RpoS. Consistent with our findings, divergent regulatory effects of RpoS and Crp‐cAMP during carbon limitation have been reported previously (Franchini *et al*., 2015). Besides RpoS, the other TFs identified not responding to Crp‐cAMP (Table 1) may be good candidate signalling regulators.

Based on our observations, we emphasize the fact that the response of cell to carbon scarcity is not limited to maintaining sufficient carbon source catabolism by Crp‐cAMP (Hua *et al*., 2004; Liu *et al*., 2005) but that its main response is to adjust metabolism to cope with slow growth rate, by decreasing the general rates of biosynthesis (Erickson *et al*., 2017). This may be extremely interesting to explore the similar situation of the Crp‐cAMP complex in other gammaproteobacteria such as *Pseudomonas putida*, where Crp‐cAMP do not appear to have any role in the control of carbon metabolism (Milanesio *et al*., 2011; Green *et al*., 2014).

Despite more than three decades of intense study, the role of ncRNAs remains somewhat elusive (Bossi and Figueroa‐Bossi, 2016). The DEGs of ncRNA genes delineated here (Fig. 1H) may account for a regulatory role acting on some of the genes not responding to Crp‐cAMP, and it will be important to explore their contributions during carbon limitation. Remarkably, the knowledge of gene expression patterns was able to uncover gene regulation mechanisms (Fig. 8). It would be interesting to explore the possible links between gene expression pattern and regulation mechanism under other physiological conditions.

A limitation of the present study is that it was developed under aerobic conditions. It is known that in the absence of oxygen the carbon source supply must be much higher than in its presence and that the overall growth rate is slower. The fact that we found growth‐responding genes such as *frdABCD*, which are coding for proteins essentially involved in growth in the absence of oxygen, suggest that by itself the growth rate could be used as a control of gene expression allowing the cell to adapt without resorting to environment‐specific signals. Further work will clarify this point.

In summary, we have described an integrative analysis of comparative transcriptome data. This systemic approach enabled us to comprehensively understand cell response to carbon limitation, quantify the contribution of Crp‐cAMP, growth rate and other factors as RpoS to the regulation of both CDS and ncRNA genes and discover global regulation strategies in *E. coli*.

## Materials and methods

### Bacterial strains and constructions

The *E. coli* strains used in this transcriptome study are the wild‐type *E. coli* K‐12 strain NCM3722 (You *et al*., 2013) and its derivative CY104, a cAMP signalling deletion mutant. CY104 was constructed by P1*vir* transduction with the lysate of strain CY105 (*crp*::*cat*), in which a *cat* allele from pBSK‐lcml was PCR amplified and integrated into the NCM3722 chromosome to replace the ORF of the *crp* gene by using the λ Red system (Datsenko and Wanner, 2000), into the recipient *cyaA*‐null strain NQ93 (You *et al*., 2013). For overexpression of the Crp protein, wild‐type *crp* from NCM3722 was cloned into pET28a via *Nde* I/*Hin*d III insertion and transformed into strain BL21 (DE3) to get strain CY134.

### Growth of batch culture

Unless specified, batch cultures were grown in N^−^ C^−^ minimal medium (You *et al*., 2013) supplemented with 22 mM glucose and 20 mM NH_4_Cl. All batch culture growths were carried out in three steps in a 37°C water bath shaker as described previously (You *et al*., 2013).

### Nutrient‐limited chemostat

A nutrient‐limited chemostat was operated using an INFORS Multifors chemostat system and performed as described previously, with slight modifications (You *et al*., 2013). The medium was N^−^ C^−^‐based, with 2.5 mM glucose and 20 mM NH_4_Cl as low‐carbon and high‐nitrogen source for the carbon‐limited chemostat, or with 11 mM glucose and 2 mM NH_4_Cl as high‐carbon and low‐nitrogen source for the nitrogen‐limited chemostat. The chemostat parameters were as follows: culture volume, 500 ml; temperature, 37 ± 0.2°C; pH, 7.0 ± 0.1; agitation, 400 ± 1 rpm; and aeration, 0.5 l/min. The initial dilution rate limiting the growth rate was set at 0.2 h^−1^, and dilution rates were respectively adjusted to 0.4, 0.6 or 0.9 h^−1^. Samples were taken for RNA extraction or viability assay after 10 generations of cell growth at a designated growth rate.

### RNA extraction

Seven millilitres of cells from chemostat growth of NCM3722 or from batch culture growth of CY104 at an exponential phase with OD600 = ~0.4 were mixed immediately with 7 ml precooled quenching buffer (60% Methanol, 70 mM HEPES) at −80°C and collected by centrifugation at 4°C. Cell pellets were resuspended in 100 μl lysis buffer [10% glucose, 12.5 mM Tris (pH 7.6), 10 mM EDTA (pH 8.0), 200 U ml^−1^ RNase inhibitor (Applied Biosystems, Waltham, MA, USA) and 40 mg ml^−1^ lysozyme (Sangon, Shanghai, China)] and treated for 3–5 min at RT. Then, 1 ml RNAiso Plus (Takara, Dalian, China) was added and followed by twice extraction with chloroform. Supernatant of extraction was then mixed with four times volume of 100% ethanol and purified by PureLink miRNA Isolation Kit (Invitrogen, Waltham, MA, USA). RNA eluted was further treated with Turbo DNase (Ambion, Waltham, MA, USA), then quantified and qualified by NanoDrop 2000 (Thermo, Waltham, MA, USA) and 2100 Bioanalyzer (Agilent, Santa Clara, CA, USA).

### Transcriptome analysis by RNA‐Seq

Strand‐specific RNA sequencing was performed. rRNA was removed with Ribo‐Zero rRNA Removal Kit (Bacteria; epicentre). mRNA was sheared to short fragments by adding fragmentation buffer, and first‐strand cDNA was synthesized with random hexamer and reverse transcriptase. The second‐strand cDNA was synthesized by adding GEX second‐strand buffer, dNTP mix, RNase H and DNA polymerase I. cDNA fragments were then purified. This step was followed by end reparation using T4 DNA polymerase and Klenow DNA polymerase. Fragments were adenylated at their 3′ ends and ligated with sequencing adapters by T4 DNA ligase. Second‐strand cDNA was degraded by UNG enzyme and purified. cDNA templates with adapters were next enriched by PCR amplification and purification. The library was sequenced using Illumina HiSeq 2000, PE125. Raw reads were filtered, and adaptors were trimmed. Clean reads were mapped to the NCM3722 genome (NCBI accession number for chromosome: CP011495.1, for plasmid F: CP011496.1) using Bowtie2. RPKM (reads per kilo bases per million mapped reads) method (Mortazavi *et al*., 2008) was used to calculate expression of each gene. Three independent RNA‐Seq assays were performed for growth of NCM3722 in carbon‐limited chemostat and for batch culture growth of CY104 in glucose. Transcriptomes from NCM3722 were analysed by ANOVA to identify genes that were statistically differentially expressed among the four growth conditions (*F*‐value> 4.07 or *P*‐value < 0.05). The genes retained by ANOVA were further analysed by *t*‐test to characterize the DEGs between 0.2 h^−1^ transcriptome and 0.9 h^−1^ transcriptome (twofold cut‐off and *P*‐value < 0.05). Two independent RNA‐Seq assays were performed for nitrogen‐limited conditions. DEGs with 1.5‐fold cut‐off and *P*‐value < 0.05 during nitrogen limitation were identified by DESeq2 (Love *et al*., 2014) software.

### Gene expression pattern analysis

The RPKM of each DEG at 0.2 h^−1^ was normalized to 1, and RPKM of each DEG at 0.4, 0.6 or 0.9 h^−1^ was determined as a ratio relative to this value. Relative expression of each DEG at each growth rate was log2‐transformed and then clustered using the Short Time‐series Expression Miner software (Ernst and Bar‐Joseph, 2006). The clustered expression patterns with *P*‐value < 0.05 were significant enriched profiles.

### 5′ RACE assay

The 5′ end of an RNA transcript was identified following the instruction of SMARTer RACE 5′/3′ Kit (Clontech). RACE‐ready cDNA was generated with random primers and SMARTer II A Oligonucleotide using 1.0–10 μg of total RNA from mid‐log cells of NCM3722 grown in glucose as template. Then, the 5′‐end DNA fragment of each targeted gene was amplified by PCR using the universal primer from the kit and gene‐specific primer (Table S1). PCR fragment with expected size was further subcloned into T vector pMD19 simple (Takara), and transcriptional start site of each gene was determined by sequencing three to nine colonies.

### Purification of Crp protein

An overnight culture of strain CY134 was diluted 1:100 into fresh LB broth plus kanamycin (50 μg ml^−1^) and grown to logarithmic phase at 37°C. One mM IPTG was added, and the culture was grown at 30°C overnight for Crp overexpression. Cells were harvested, and N‐terminal His‐tagged Crp was purified following the instruction of Capturem His‐Tagged Purification Miniprep Kit (Takara). Crp protein with purity larger than 95% was used for EMSA study.

### EMSA of Crp‐cAMP binding to promoters

The promoter region of each gene was PCR amplified and purified. DNA‐binding reaction by Crp‐cAMP was set up as follows: 50 nM DNA, 0–1200 nM Crp proteins in binding buffer [10 mM Tris–HCl (pH 7.2), 150 mM NaCl, 3 mM MgAc, 1 mM cAMP and 5% glycerol]. The reaction mix was incubated at 37°C for 15 min and separated by electrophoresis on 6% native polyacrylamide gel at 120 V for 60 min. Then, gel was stained and visualized. Two to six independent repeats were performed for each electrophoretic mobility shift assay.

### Viability assay

To test cell's viability to oxidative stress, cells grown in carbon‐limited chemostat at a growth rate of 0.2  and 0.9 h^−1^ were respectively challenged with 10 mM hydrogen peroxide for 60 min. Unchallenged cells at each growth rate were set as controls. Cells were spread on LB plate, incubated at 37°C overnight, and CFUs were counted. Viability was calculated as the percentage of CFUs of cells challenged with stress versus unchallenged cells. Independent biological duplicates were performed.

## Conflict of interest

None declared.

## Supporting information


**Fig. S1.** Expression changes of genes enriched in the 10 non‐significantly enriched profiles following growth rates.

**Table S1.** Transcriptional start site (TSS) of new promoters identified.
**Table S2.** Prediction of Crp‐cAMP binding site at the promoter region of corresponding genes.

**Data S1.** DEGs identified during carbon limitation.

**Data S2.** Relative expression of DEGs and their expression patterns following growth rates during carbon limitation.

**Data S3.** Six groups of genes with various regulatory mechanisms.

**Data S4.** Genes subjected to GRDC and controlled by other regulators which respond to nitrogen limitation.
