# Supplementary material for: Deciphering global gene expression and regulation strategy in Escherichia coli during carbon limitation
Source: Microb Biotechnol. 2018 Dec 11;12(2):360–76. doi: 10.1111/1751-7915.13343 (PMC6390033; doi:10.1111/1751-7915.13343)
Supplement: Supplementary file 2 — Table S1. Transcriptional start site (TSS) of new promoters identified. Table S2. Prediction of Crp‐cAMP binding site at the promoter region of corresponding genes. [file MBT2-12-360-s002.pdf]

Table S1. Transcriptional start site (TSS) of new promoters identified.

Criterion	Gene	Position[a]	TSS	Putative promoter (starting from -1bp relative to TSS)[b]	Location of promoter	Sigma factor
1	*allB*	-106	A	ATCGTCGCCTTTGCGGCCTACGCCT**TATTAA**AGA	ybbW coding region	Sigma70
		-161	G	CGGTAAGTTTATTCACTTTATGGAAC**CGTTAT**CGCGT	ybbW coding region	Sigma70
		-203	C	**TTTTCA**GTAACTCTGGTGGCCGT**TATTTT**ATCT	ybbW coding region	Sigma70
	csgA	-211	G	**CAGACA**GGAGATTATAACCTTGCA**TATATT**GATCAG	csgB coding region	Sigma70
	dmsC	-76	G	**CCGTTG**CCGCGAGCTCACTTTACCAAA**CCGAAT**ATT	dmsB coding region	Sigma70
	frlR	-9	G	**ACGACC**CTATAATCCGAGTAATTCATTC**TTTATT**TCA	Intergenetic region	Sigma70
		-19	C	**TTGCCG**GAAAACGACCCTATAATCCG**AGTAAT**TCATT	Intergenetic region	Sigma70
	fucO	-210	G	**CTCTCA**AAAATCGTAAGGCAACTTTGT**TACAAC**ATCAT	fucA coding region	Sigma70
	fucU	-51	A	AGCCCGGAAGAAGCCCGCGCACAGATT**CATTAT**CAGT	fucK coding region	Sigma70
	garR	-69	C	GAAGCCGATGCGCGTCGTTATCTGGAATGGGGCGCGACGTTTGTGGCTGTCGGCAGCGATCTCGGCGTCTTCCGCTCTGC	garL coding region	ND
	glpQ	-113	T	GCTACACCG**TGGACT**TCTTCG**GCTGG**GATGGCGGCTT	glpT coding region	Sigma54
	gnsA	-63	G	TTCGGGGAGA**TGGCTT**ATAACA**TTTCT**TACCTGACCA	ymcE coding region	Sigma54
		-68	G	**TTGAAT**CTTTCGGGGAGATGGCTTATAACA**TTTCTT**ACCT	ymcE coding region	Sigma70
	mngB	-153	G	GCATTTTCTCGCTCTTTTTACTTCAT**GATAAT**GGCGC	mngA coding region	Sigma70
		-183	C	TTTGTGCTGGGCTCTATGGTAACGGGCGCTATTGTCGGTGCGATGAATATCGGCCTTTCGACACCCGGTGCCGGCATTTT	mngA coding region	ND
	paaJ	-92	G	TTTTGCGTCCAGGCTTTGCCGGAGACACCTTAACTGCTACTGCGCAGGTACGTCATCAGGGCAAGCAAACCGGTGTTTAC	paaI coding region	ND
		-122	C	CAGCCGTCGCTTCTGCCTGCACGATTGATTTTTTGCGTCCAGGCTTTGCCGGAGACACCTTAACTGCTACTGCGCAGGTA	paaI coding region	ND
		-249	A	GGCTTTGCTGTAGTGACCATGACCGTCACTGCACAAATGCTTAACGGTCATCAAAGTTGCCACGGCGGGCAGCTATTTTC	paaI coding region	ND
	yjhC	-21	G	**TTGATA**TTCCAGGTAAAATTTATA**AACTAT**CCGT	yjhB coding region	Sigma70
		-27	A	**TTGGTT**TTGATATTCCAGGTAAA**ATTTAT**AAACT	yjhB coding region	Sigma70
	xapB	-266	G	**GAGACT**GCGGCGGAAATTCGCATGATGCA**AATTAT**TGGT	xapA coding region	Sigma70
		-318	G	GTTTCCCTCTGACGGAGGGCGTGTTCG**TCTCGT**ATCC	xapA coding region	Sigma70
		-390	C	GCTTCTTCTCAC**TGGCAA**ATGCCT**ACGAT**GCGGAATA	xapA coding region	Sigma54
2	*btuD*	-25	G	**CGGATA**TGACGCCGGAAGATCCCAT**TGTGAT**GGAAA	btuE coding region	Sigma70
	btuE	-15	C	**GGTCTA**TGATTAAAAGCTAAGATT**TTTACT**GACCA	Intergenetic region	Sigma70
		-20	G	**AAGACG**GTCTATGATTAAAAGCTAA**GATTTT**TACT	Intergenetic region	Sigma70
	malY	-117	A	**TTGTTA**ATGTGCAGGCACTGAAG**GACAAT**CGGGCA	malX coding region	Sigma70
	psiF	-50	A	**GTGGTT**TTTTTGCTGTTAGCAACCAGA**CTTAAT**GGC	Intergenetic region	Sigma70
	speD	-135	A	**CAGATA**ACGACGCCTTACGCCATCTC**TCAACC**GAAATT	speE coding region	Sigma70
	speE	-40	G	**TTGACC**GTGCATCCGCGGTCAATGTTAGC**TATTAT**GTTGC	Intergenetic region	Sigma70

1&2	*aroM*	-28	G	**TCAGCA**GTGGTGCTTCACACTTGCCC**GGTAAT**TAAC	Intergenetic region	Sigma70
	lpxT	-28	G	AAGCTTCGTTTAGCGACTACCGCG**TAAGGT**TGCCTGC	yeiR coding region and intergenic region	Sigma70
	nei	-31	G	**TTGCCG**AAAAGGGGATTGTTGTCGCAG**CATAAC**CCC	pxpA coding region and intergenic region	Sigma70
	uhpB	-158	G	GCGGCTGTTATCTGACGCCGGATATTGCCATTAAACTGGCATCCGGTCGTCAGGACCCGCTAACCAAACGTGAACGCCAG	uhpA coding region	ND

TSS: transcriptional start site

ND: Not determined

[a]Relative to translational start site

[b]The predicted -35 and -10 region of sigma 70 factor, or -24 and -12 region of sigma 54 factor was in bold.

Table S2. Prediction of Crp-cAMP binding site at the promoter region of corresponding gene.

Gene	Start position[a]	End position[a]	Sequence
hcaR	-60	-39	ATATGTGAAATAGTTAATTTCT
rspA	-101	-80	AAAAGCCGGAGTGATCACAAAA
yqeF	-91	-70	CTTTGTGATAAAAATCACTTTT
yahN	-41	-20	CTTTGTGATCTCTCTCGCACCC
psuK	-230	-209	TTCTGTGCATCCCGTCACAAAT
rihA	-44	-23	CTTTGCGACAAGGGTAACGCCA
mokB	-212	-191	ATTCGTTACTCTCTTCATTCCA
ygeW	-168	-146	ATTTGTGATCAACCCCACACAT
ygcW	-200	-180	AAGCGTGATTACTCTCACACAA
yrbN	-26	-5	AAGTGTGAACCGGCTCAAAGTA
ydcX	-69	-48	AAACTTGATGCACGTCAAAAAA
cdaR	-109	-88	ATCTGTGAACTTCACCACAACT
ddpX	-200	-179	TACTGTGATTTCAGCTACGATT
bsmA	-64	-43	AATCGCGTACCAGGTAACAATT
sgcX	-87	-66	TCACGTGAACGTTCTCACAATT
abgR	-153	-132	AAGTGTGACCCGGTTCACGTAG
xdhA	-152	-141	TTTTTTTAACGTCATCACACTT
fadI	-112	-91	AAGTGGGAGCTATGACACAGAG
arcZ	-212	-191	CTGCGTGAAATAGCTAACAACG
ygeV	-216	-194	AAAGGGGCAGGCTATCTCTCTT

[a] Relateive to translational start site

Basing on the results of EMSA, we analyzed the promoter sequence by Virtual Footprint (http://www.prodoric.de/vfp/vfp_promoter.php) and predicted Crp-cAMP binding site of each gene.
