# Supplementary material for: Deciphering global gene expression and regulation strategy in Escherichia coli during carbon limitation
Source: Microb Biotechnol. 2018 Dec 11;12(2):360–76. doi: 10.1111/1751-7915.13343 (PMC6390033; doi:10.1111/1751-7915.13343)
Supplement: Supplementary file 1 — Fig. S1. Expression changes of genes enriched in the 10 non‐significantly enriched profiles following growth rates. [file MBT2-12-360-s001.pdf]

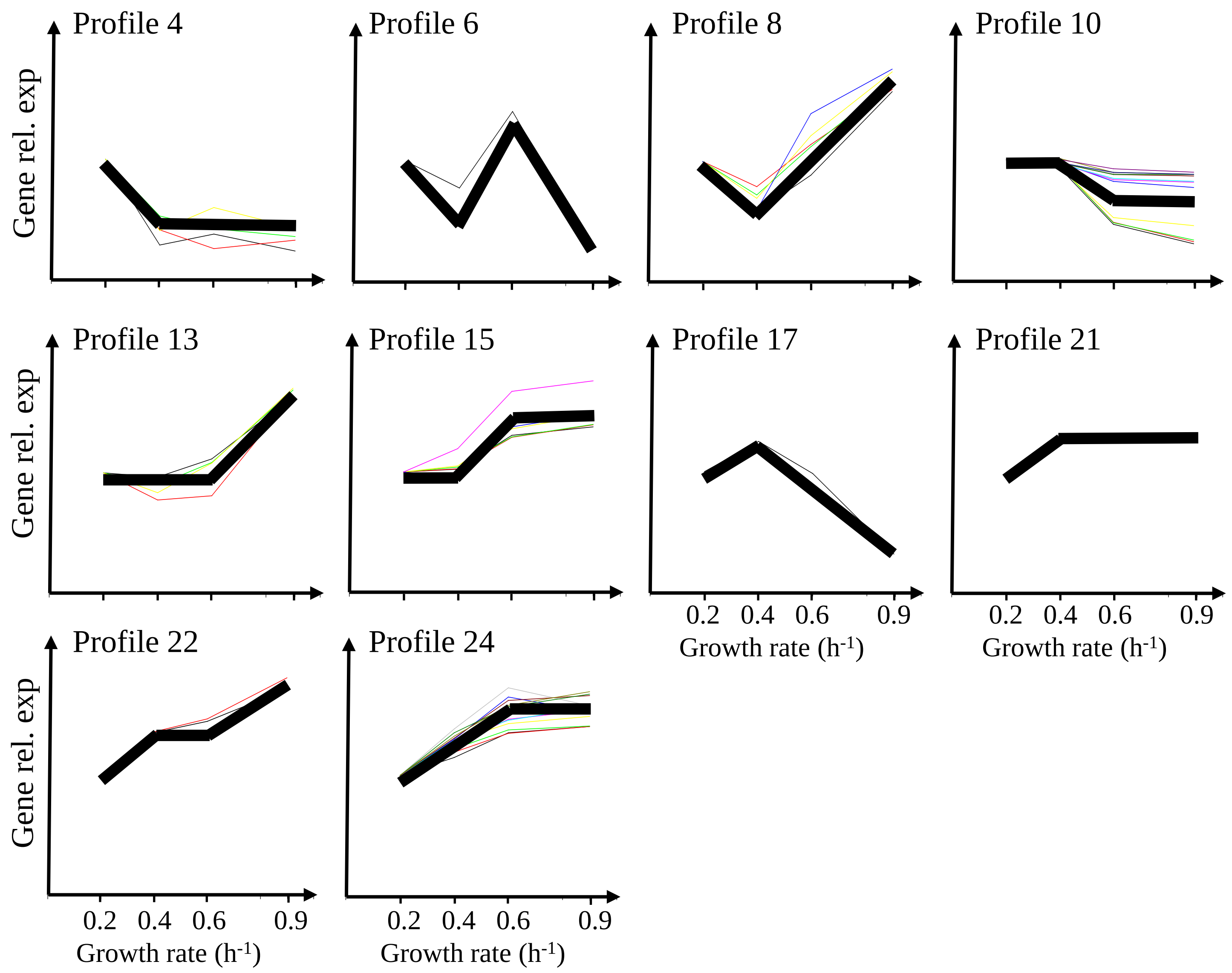

Profile 4
Profile 6
Profile 8
Profile 10
Profile 13
Profile 15
Profile 17
Profile 21
Profile 22
Profile 24
Gene rel. exp
Gene rel. exp
Gene rel. exp
0.2 0.4 0.6 0.9
Growth rate (h-1)
0.2 0.4 0.6 0.9
Growth rate (h-1)
0.2 0.4 0.6 0.9
Growth rate (h-1)
0.2 0.4 0.6 0.9
Growth rate (h-1)
